# Managing Side Effects of Cancer Patients Treated With Immunotherapy

**Published:** 2018-04-01

**Authors:** Brianna Hoffner, Laura J. Zitella

**Affiliations:** 1 University of Colorado, Aurora, Colorado;; 2 Stanford Cancer Institute, Stanford, California

## Abstract

Immunotherapy has proven activity in several types of cancer; however, the mechanisms of action of immunotherapy can cause several different immune-related toxicities. Advanced practitioners are in a unique position to recognize the timing and manage these toxicities to help patients realize the maximal clinical efficacy of their therapy.

In a span of fewer than 7 years, immunotherapy has transformed the therapeutic landscape of oncology. Beginning with ipilimumab (Yervoy) in 2011, the FDA has thus far approved six immunotherapeutic agents (immune checkpoint inhibitors) for oncology indications, including five drugs that target programmed cell death protein 1 (PD-1) or its ligand (PD-L1). Immunotherapy has proven activity in no fewer than a dozen types of cancer, including solid tumors and hematologic malignancies (Champiat et al., 2016; Chiou & Burotto, 2015). The list of approved drugs and indications will likely expand in the near future, as ongoing investigations evaluate the activity of existing and investigational immunotherapeutic agents in multiple types of cancer. However, the mechanisms of action that achieve wide-ranging antitumor activity can cause a host of immune-related toxicities. Effective management of these toxicities plays a key role in reaching and maintaining maximal clinical efficacy, as discussed at JADPRO Live 2017 by Brianna Hoffner, MSN, RN, ANP-BC, AOCNP®, of the University of Colorado, and Laura J. Zitella, MS, RN, ACNP-BC, AOCN®, of Stanford Cancer Institute.

## PATHOPHYSIOLOGY AND GENERAL APPROACH

The CTLA-4 pathway, targeted by ipilimumab, and the PD-1 pathway are associated with immune tolerance. The wide-ranging activity of immune checkpoint inhibitors results from the drugs’ activation of the human immune system, a generic type of antitumor activity, as opposed to induction of a disease-specific anticancer activity. Early clinical studies of immune checkpoint inhibitors often involved cancers that exhibited minimal susceptibility to conventional chemotherapy. Inhibition of the immune checkpoint pathways removes tolerance, a phenomenon often described as "releasing the brakes on the immune system." Removing tolerance also leads to activation of the immune system against cancer cells.

"At the same time, if we activate the immune system so that you lose tolerance, sometimes normal cells, which should have tolerance to the immune system, suddenly become targets of the immune system," said Ms. Zitella.

Most adverse effects associated with immune checkpoint inhibitors are mild or moderate, and the most common toxicities are rash and diarrhea. However, less common and potentially severe toxicities can occur (Champiat et al., 2016), and the list continues to grow as indications and clinical experience with immune checkpoint inhibitors increase (Table 1).

**Table 1:**
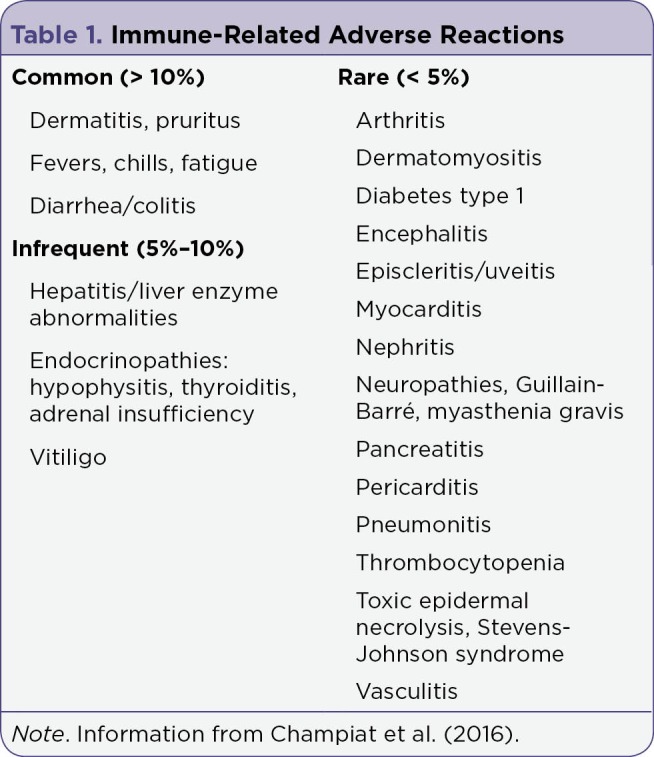
Immune-Related Adverse Reactions

Immune-related side effects do not occur immediately, but instead emerge over the course of several weeks of treatment, especially with the PD-1/PD-L1 inhibitors (4–10 weeks). CTLA-4 inhibition produces more severe side effects, which tend to occur earlier in the course of treatment. Similarly, combination therapy with a CTLA-4 inhibitor and a PD-1/PD-L1 inhibitor causes more severe side effects that appear earlier.

The European Society for Medical Oncology published guidelines on screening patients for the risk of immunotherapy-associated toxicities (Champiat et al., 2016), and both the National Comprehensive Cancer Network (NCCN) and the American Society of Clinical Oncology have released their own consensus guidelines (Brahmer et al., 2018; NCCN, 2018), said Ms. Zitella.

In the absence of consensus, the management of immune-related adverse events remains empiric. For mild or moderate (grade 1–2) side effects, supportive care is indicated, whereas severe side effects (grade 3–4) are usually treated with steroids. The principal exceptions pertain to hypothyroidism and other endocrinopathies, which might be treated with hormone replacement therapy. In those cases, steroids will be omitted.

Use of steroids introduces several other considerations (NCCN, 2017). Patients who require steroids, particularly high-dose steroids, often develop gastritis, which can be prevented with a histamine receptor antagonist (H2 blocker). Many patients on steroids develop oral thrush, which can be prevented with good oral care and the use of clotrimazole troches. Finally, patients treated with prednisone at 20 mg or equivalent for more than 4 weeks should receive pneumocystis prophylaxis (*Pneumocystis jirovecii*) with sulfamethoxazole/trimethoprim or an alternative.

Rarely, patients develop steroid-refractory immune-related adverse events. One of two drugs is used in most cases: infliximab and mycophenolate mofetil (Friedman, Proverbs-Singh, & Postow, 2016). Patients with a history of hepatitis should receive mycophenolate mofetil, as infliximab may be hepatotoxic.

If a patient discontinues immunotherapy because of side effects, rechallenging is an option.

"In general, our approach is that if the side effect resolves and the steroid dose is reduced to less than 10 mg per day of prednisone, or the equivalent, and the patient is not on any other immunosuppressive drugs, then it is okay to restart the immunotherapy," said Ms. Zitella. "Keep in mind, we have seen in our practice that patients may continue to benefit from the immunotherapy long after you have discontinued it."

## DIAGNOSIS AND MANAGEMENT OF SELECTED IMMUNE-RELATED ADVERSE EVENTS

**Anemia**

Standard laboratory diagnosis of anemia includes hemoglobin (Hgb, 14.3–18.1 g/dL), mean corpuscular volume (MCV, 80–100 fL) and reticulocyte count (0.5%–2.5%). To determine if there is iron deficiency anemia, one can add on transferrin saturation and ferritin (11–307 ng/mL).

For patients with Hgb < 12 g/dL (women) or Hgb < 13 g/dL (men), stratification begins with the reticulocyte index. An index < 2, associated with MCV > 100 fL, is diagnostic for hypoproliferative macrocytic anemia. If the MCV ranges between 80 and 100 fL, the patient has hypoproliferative normocytic anemia. An MCV < 80 fL, in association with a reticulocyte index < 2, is indicative of hypoproliferative microcytic anemia. A reticulocyte index > 2 is diagnostic for acute blood loss hemolysis, irrespective of the MCV (Camaschella, 2015; Table 2).

**Table 2:**
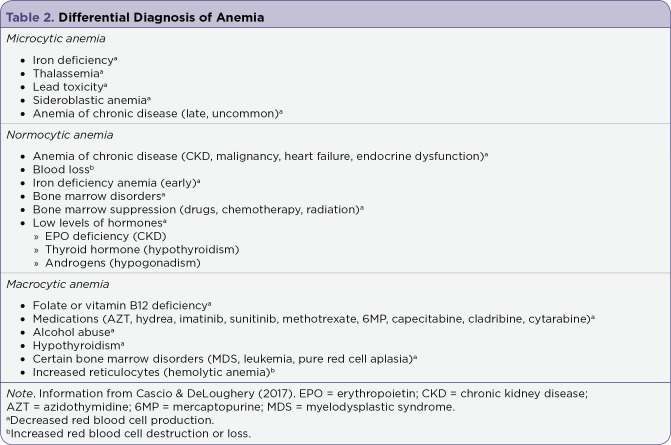
Differential Diagnosis of Anemia

Anemia, especially normocytic anemia, can have multiple causes, most of which lead to decreased red blood cell production (Cascio & DeLoughery, 2017). Anemia due to increased red blood cell destruction or loss has two prominent causes: blood loss (normocytic anemia) or hemolytic anemia (normocytic or macrocytic anemia). Bone marrow suppression, which can cause normocytic anemia, is common among patients with cancer, as chemotherapy, radiation, and other drugs used to treat cancer often cause myelosuppression.

"It is a hard thing because often anemia is multifactorial, but we have to do really good investigative work to come up with the best answer possible," said Ms. Hoffner.

**Elevated Creatinine**

Elevated creatinine is not uncommon in patients with cancer, and multiple causes have to be considered. An uncommon cause that should not be overlooked in patients receiving immunotherapy is autoimmune nephritis (Cortazar et al., 2016; Haanen et al., 2017; Hassel et al., 2017; Wanchoo et al., 2017). The incidence of treatment-induced acute kidney injury and subsequent nephritis increases with combination immunotherapy, such as ipilimumab plus a PD-1/PD-L1 inhibitor, said Ms. Hoffner. The onset of nephritis occurs earlier with ipilimumab (2–3 months) as compared with PD-1/PD-L1 inhibitors (3–10 months). Onset also tends to be sooner with combination therapy.

Most patients who develop immunotherapy-induced nephritis recover renal function with steroid therapy. Recovery of renal function usually occurs after several weeks, and steroids should be tapered gradually beyond renal recovery to avoid a rebound phenomenon, said Ms. Hoffner.

**Autoimmune Hyperthyroidism**

Subclinical hyperthyroidism is associated with low thyroid-stimulating hormone (TSH) and normal free T4, whereas primary hyperthyroidism is associated with low TSH and elevated free T4. Radioactive iodine uptake usually is inaccurate for a diagnostic test. Antithyroglobulin and antithyroid peroxidase lab results are usually positive. In most cases, autoimmune hyperthyroidism is asymptomatic, but patients with symptomatic hyperthyroidism can be managed with a beta blocker. Delaying immunotherapy is an option, to allow time for thyroid function to return to normal. The condition generally is self-limiting but can be distressing to patients (Byun, Wolchok, Rosenberg, & Girotra, 2017).

"The data has shown us that oftentimes patients who we diagnosed with hypothyroidism have previously been hyperthyroid, and we missed that time period," said Ms. Hoffner. "They go up and they burn out and they come back down. If you catch them in that hyperthyroid time period, they can actually be quite sick. I have had patients who presented in atrial fibrillation with rapid ventricular response and looking sick, so get your endocrine colleagues involved to help you with that."

**Hypophysitis**

Inflammation of the pituitary gland arises from a deficiency involving some or all of the pituitary hormones. The symptom constellation comprises headache, fatigue, muscle weakness, constipation, cognitive difficulties, erectile dysfunction/amenorrhea, and orthostatic hypotension. The standard workup consists of a complete pituitary lab panel (adrenocorticotropic hormone, TSH, follicle-stimulating hormone, luteinizing hormone, and cortisol) and a contrast magnetic resonance imaging brain scan with pituitary cuts (Byun et al., 2017; Michot et al., 2016).

High-dose steroid therapy is indicated for critical illness. Low-dose glucocorticoids can alleviate headache and fatigue. Patients should receive hormone replacement therapy for any deficiency.

**Diarrhea/Colitis**

Symptoms are straightforward: abdominal cramping, pain, anorexia, dyspepsia, diarrhea, and blood or mucous in stool. "A patient can have colitis without diarrhea, and I think we didn’t fully realize or appreciate that," said Ms. Hoffner. "It’s similar to those very rare cases where you have a patient who has *Clostridium difficile* but doesn’t have diarrhea, and it just doesn’t make sense. We see that here, as well. In your workup, always rule out infectious causes."

In addition to stool and blood analyses, contrast computed tomography of the abdomen and pelvis can reveal any thickening of the bowel that would be characteristic of colitis.

Ms. Hoffner emphasized that the occurrence of diarrhea and/or colitis with one type of immune checkpoint inhibitor does not rule out treatment with a different checkpoint inhibitor.

**Pneumonitis**

About 1% to 2% of patients treated with a PD-1/PD-L1 inhibitor or CTLA-4 inhibitor develop inflammation of the lining of the lungs. Pneumonitis tends to occur after 9 to 19 weeks of treatment. Symptoms are unremarkable: dry, unproductive cough; dyspnea; cyanosis; and fatigue. The differential diagnosis should include infection, allergies, and potential cardiac causes, such as pericarditis (Michot et al., 2016). It is important for advanced practitioners to keep in mind that a late diagnosis of pneumonitis can result in chronic, irreversible lung disease (Eigentler et al., 2016).

**Diabetes Mellitus**

Rarely (< 0.1%), a patient treated with a PD-1/PD-L1 inhibitor will develop type 1 diabetes mellitus. When diabetes occurs, patients generally have diabetic ketoacidosis at presentation (Byun et al., 2017). The workup should include a glutamic acid decarboxylase (GAD65) antibody test. A positive GAD65 is indicative of autoimmune diabetes. Patients usually respond to insulin therapy, but an endocrinology consult is essential.

**Oral Mucosa**

The symptom spectrum is fairly limited: mucositis, gingivitis, and sicca (Sjögren) syndrome. Lab work should include a screen for antinuclear and Sjögren syndrome–related antigen A/Sjögren syndrome–related antigen B antibodies. Treatment options include a corticosteroid rinse, pilocarpine chlorhydrate, viscous lidocaine, in addition to good oral hygiene (Friedman et al., 2016; Michot et al., 2016).

**Pancreatic**

The spectrum of signs and symptoms encompasses asymptomatic elevation in amylase/lipase and pancreatitis. The clinical relevance of asymptomatic elevations in amylase or lipase remains unclear (Friedman et al., 2016).

**Neurologic**

Neurologic immune-related adverse events occur in fewer than 5% of patients treated with immune checkpoint inhibitors. Adverse effects include neuropathies, aseptic meningitis, temporal arteritis, myasthenia gravis, and Guillain-Barré syndrome. Steroid treatment is commonly used but not universally effective. Some patients may require treatment with intravenous immunoglobulin (Friedman et al., 2016).

**Polyarthritis/Arthralgia**

These conditions arise in about 5% of patients treated with immune checkpoint inhibitors. Anecdotally, the condition may be underreported, said Ms. Hoffner. In most cases, the condition and symptoms are mild or moderate, and most patients respond to low-dose steroids. Occasionally, more serious conditions arise, such as lupus erythematosus (Michot et al., 2016).

**Hematologic**

As previously described, anemia has been reported in fewer than 5% of patients treated with a CTLA-4 inhibitor and fewer than 10% of patients treated with a PD-1/PD-L1 drug (Friedman et al., 2016). Other hematologic immune-related adverse events that have been reported include red cell aplasia, autoimmune neutropenia, pancytopenia, and acquired hemophilia (Michot et al., 2016). The workup should include a peripheral smear, reticulocyte count, Coombs test, hemolysis assays, and bone marrow biopsy.

Recent case reports have highlighted additional immune-related adverse events that affect cancer patients treated with immune checkpoint inhibitors. One report described fulminant myocarditis that occurred in patients with melanoma treated with the combination of ipilimumab and nivolumab (Johnson et al., 2016). The other described cerebral vasculitis in patients with lung cancer treated with a PD-1 inhibitor (Laubli et al., 2017).

The cases illustrate how "Anything is possible," said Ms. Hoffner. "We must remember that when we take care of these patients."
